# Prof. Smith’s Laboratory

**Published:** 1854-11

**Authors:** 


### PROF. SMITH'S LABORATORY.

Professor J. Lawrence Smith has fitted up in this city, at his own expense, a laboratory of research, and his advertisement will be found upon the cover of this journal. Such a laboratory has been very much needed in Louisville, and we are confident Prof. Smith will soon have as much work in the shape of assays and analyses as he can find time to accomplish. We need hardly inform our readers that he has had great experience in this department of science, and that those who engage his services will find him in his investigations thorough, accurate, and reliable. He offers also to instruct a few students in chemical manipulation and analysis. The opportunity is a favorable one for those who wish to make themselves practical chemists, and the number of such, considering the demand for teachers of chemistry in our country, ought not to be inconsiderable.

